# Genetic Characterization of Lumpy Skin Disease Viruses Circulating in Lesotho Cattle

**DOI:** 10.3390/v16050762

**Published:** 2024-05-11

**Authors:** Mabusetsa Joseph Raporoto Makalo, Tirumala Bharani Kumar Settypalli, Irene Kasindi Meki, Mame Thierno Bakhoum, Hatem Ouled Ahmed, Moeketsi Solomon Phalatsi, Tsepo Ramatla, ThankGod Emmanuel Onyiche, Lineo Nionzima-Bohloa, Artem Metlin, Madhur Dhingra, Giovanni Cattoli, Charles Euloge Lamien, Oriel Matlhahane Molifi Thekisoe

**Affiliations:** 1 Unit for Environmental Sciences and Management, North-West University, Potchefstroom 2531, South Africa; ra21205450@gmail.com (T.R.); et.onyiche@unimaid.edu.ng (T.E.O.); oriel.thekisoe@nwu.ac.za (O.M.M.T.); 2 Department of Livestock Services, Ministry of Agriculture, Food Security, and Nutrition, Private A82, Maseru, Lesotho; lineo.bohloa@gmail.com; 3 Animal Production and Health Laboratory, Animal Production and Health Section, Joint FAO/IAEA Division, Department of Nuclear Sciences and Applications, International Atomic Energy Agency, P.O. Box 100, 1400 Vienna, Austria; t.b.k.settypalli@iaea.org (T.B.K.S.); i.meki@iaea.org (I.K.M.); ouledahmedh@gmail.com (H.O.A.); g.cattoli@iaea.org (G.C.); c.lamien@iaea.org (C.E.L.); 4 Laboratoire National de l’Elevage et de Recherches Vétérinaires ISRA/LNERV(LNERV), BP 2057, Dakar, Senegal; thierno.bakhoum@gmail.com; 5 Department of Animal Science, National University of Lesotho, P.O. Roma 180, Lesotho; moeketsiphalatsi@gmail.com; 6 Department of Veterinary Parasitology and Entomology, University of Maiduguri, P. M. B. 1069, Maiduguri 600230, Nigeria; 7 Food and Agriculture Organization of the United Nations, Viale delle Terme di Caracalla, 00153 Rome, Italy; artem.metlin@fao.org (A.M.); madhur.dhingra@fao.org (M.D.)

**Keywords:** lumpy skin disease virus, whole genome, cattle, Lesotho

## Abstract

Lumpy skin disease is one of the fast-spreading viral diseases of cattle and buffalo that can potentially cause severe economic impact. Lesotho experienced LSD for the first time in 1947 and episodes of outbreaks occurred throughout the decades. In this study, eighteen specimens were collected from LSD-clinically diseased cattle between 2020 and 2022 from Mafeteng, Leribe, Maseru, Berea, and Mohales’ Hoek districts of Lesotho. A total of 11 DNA samples were analyzed by PCR and sequencing of the extracellular enveloped virus (EEV) glycoprotein, G-protein-coupled chemokine receptor (GPCR), 30 kDa RNA polymerase subunit (RPO30), and B22R genes. All nucleotide sequences of the above-mentioned genes confirmed that the PCR amplicons of clinical samples are truly LSDV, as they were identical to respective LSDV isolates on the NCBI GenBank. Two of the elevem samples were further characterized by whole-genome sequencing. The analysis, based on both CaPV marker genes and complete genome sequences, revealed that the LSDV isolates from Lesotho cluster with the NW-like LSDVs, which includes the commonly circulating LSDV field isolates from Africa, the Middle East, the Balkans, Turkey, and Eastern Europe.

## 1. Introduction

Lumpy skin disease (LSD) is a highly contagious, transboundary, and fast-spreading viral disease causing significant economic losses in the livestock industry in Africa, parts of Europe, and Asia [1,2]. This disease is caused by LSD virus (LSDV), a double-stranded DNA virus with a genome length of 151 kbp belonging to the family *Poxviridae* Genus *Capripoxvirus*, which also comprises goatpox virus (GTPV) and sheeppox virus (SPPV) [3,4,5]. Lumpy skin disease was first discovered in Zambia in 1929 [6]. The disease later spread to the rest of the African continent (excluding Algeria, Morocco, and Tunisia), into the Middle East, Southeastern Europe, Central Asia, South Asia, Russia, Turkey, and China [7,8,9,10,11]. The disease mainly affects cattle but also wild mammalian species, including giraffes, impalas, water buffaloes, and camels [12,13,14,15,16]. In cattle, the disease is characterized by fever (39–41 °C), nodular lesions on the skin and mucus membrane, swelling of lymph nodes, and the formation of cutaneous coalescing firm nodules that develop into necrotic cores [17]. The disease causes a considerable reduction in milk production, loss of body weight, and sometimes death in cattle [18,19].

In endemic areas, LSD morbidity and mortality rate is estimated to be 10% (varies between 3% and 85%) and 1–3%, respectively [20]. Several risk factors, including low-host immunity, introduction of new animals, lack of biosecurity measures, proximity to wildlife mammalian species, especially buffalo, free movement of animals, and contact between infected animals and susceptible hosts, contribute to the spread of LSDV [21,22,23]. The transmission of LSDV is mainly by biting and blood-feeding arthropod vectors that include *Tabanidae*, *Muscidae*, and *Glossina* species [24,25,26]. Long-distance transmission, such as between farms and countries, is associated with the movement of infected asymptomatic animals and vectors during trade [27]. Currently, there is no specific cure or treatment for LSD [28]. However, agents such as ivermectin have shown therapeutic potential for the treatment of LSD in cattle [29]. While vaccination is regarded as the most effective control measure, its combination with strict animal and vector control yields better results [30].

Several DNA-based tests have been developed for detecting and differentiating LSDV strains and other Capripoxvirus species with a high level of accuracy [31,32,33]. These tests are based on detecting unique genetic markers or specific DNA sequences characteristic of LSDV strains or Capripoxvirus species [34]. By comparing these gene sequences, scientists can accurately distinguish between LSDV strains and identify LSDV from GTPV and SPPV [35,36]. Moreover, these tests can also be used to determine virulence, track and identify the origin of the LSDV, and monitor vaccine efficacy and quality [37,38]. Commonly used genetic markers, either as complete genes or partial gene sequences, include B22R, G-protein-coupled receptor (GPCR), extracellular enveloped virus (EEV) glycoprotein, P32 antigen, and RNA polymerase 30kDa subunit (RPO30) [39,40,41]. Whole-genome sequencing offers a platform for analyzing the entire genome with more resolution when compared to single-gene analysis [42,43,44]. With the advent of second- and third-generation sequencing technology, there has been an increase in research and publications on the complete genome of LSDV over the past two decades [45,46]. This provides helpful information for vaccine quality, LSDV surveillance, and research, thereby increasing knowledge on disease prevention and control [3,6,47,48].

A great deal of research and knowledge development is currently being conducted on the characterization of circulating LSDV strains based on partial and complete gene sequencing in sub-Saharan countries, including South Africa, Namibia, Botswana, and Zimbabwe [49,50,51]. Lesotho experienced the first outbreaks of LSD in cattle as early as 1947, followed by intermittent outbreaks throughout the following decades [52,53]. However, there is a lack of scientific information regarding the genetic and genomic characteristics of LSDV isolates associated with the outbreak episodes in the country. Therefore, this study sought to provide information regarding the genetic and genomic characteristics of the Lesotho LSDV isolates to improve the management of this disease.

## 2. Materials and Methods

### 2.1. Study Area

**Figure 1:**
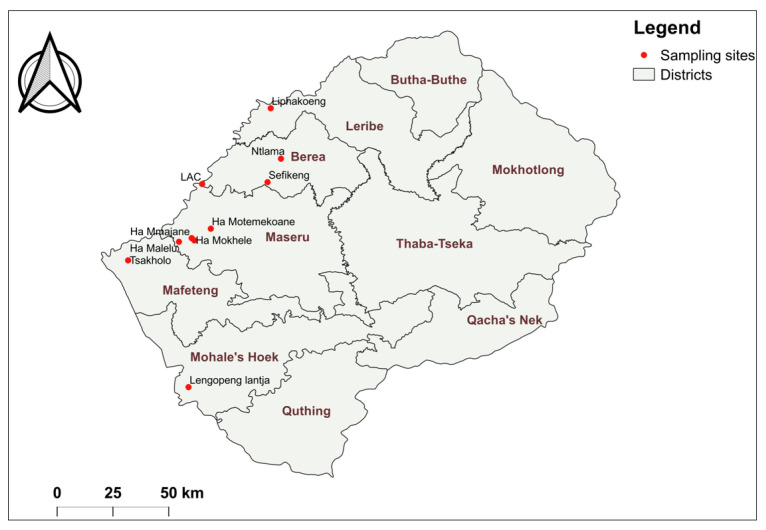
Specimen collection sites for detection and characterization of LSDV. The sampled villages are highlighted by red dots.

The study was conducted in various villages in five districts of Lesotho (Figure 1), namely Berea, Maseru, Leribe, Mohales’ Hoek, and Mafeteng, during the months of January, February, and March of 2020 and 2022. The study population comprised 18 cattle that manifested LSD clinical signs and symptoms. All sampled cattle were not previously vaccinated against LSD.

### 2.2. Sample Collection

A total of 18 skin scrapings of suspected LSD nodule signs were collected using separate specimen bottles bearing unique animal identities and transported to the Central Veterinary Laboratory for processing and analysis following necessary biosafety and biosecurity procedures. The recorded information included the place of collection, date, and coordinates (Table S1).

### 2.3. Sample Extraction and Processing

The skin scrapings were ground in 2 mL phosphate-buffered saline (PBS) and centrifuged for 10 min at 590× *g* before the extraction of DNA using an RNeasy Plus Mini Kit (Qiagen, Hilden, Germantown, MD, USA) as directed by the manufacturer. 

### 2.4. DNA Amplification

Real-time PCR was used to screen the 18 samples according to published literature [54]. A total volume of 20 μL PCR master mix containing 10 μL of BioRad iQ Supermix, 6.7 μL nuclease-free water, 0.4 μL (20 pmol/μL) of the primers CaPV074-F1: AAAACGGTATATAGAGTTAA and CaPV074-R1: AAATGAAACCAATGGATGGGGATA), 0.5 μL (10 pmol/μL) of the probe CapV074P1; Fam-TGGCTCATAGATTTCCT and 2 μL of DNA was run under the following program: initial denaturation at 95 °C for 10 min, followed by 40 cycles of 95 °C for 15 s and 60 °C for 60 s.

### 2.5. Amplification and Sequencing of Selected CaPV Genes

Four selected CaPV-marker genes (B22R, EEV glycoprotein, GPCR, and RPO30) were amplified and sequenced according to published literature using the primers shown in Table 1 [35,36,40]. The PCR products were analyzed on a 2% agarose gel in 1X TAE buffer and visualized under UV light. The PCR products were purified and sent for Sanger sequencing from both directions using forward and reverse primers at LGC Genomics (Berlin, Germany).

### 2.6. Whole-Genome Sequencing of LSDV

Whole genome sequencing was performed using Ion S5 technology, following the previously published procedure [55]. Briefly, LSDV-positive DNA (~100 ng) from two samples from Lesotho was enzymatically fragmented into 200 bp lengths using Ion shear Plus reagents, then adapters and barcodes were ligated using the Ion Xpress™ Plus Fragment Library Kit and the Ion Xpress barcode adapters (Thermo Fisher Scientific, Berlin, NH, USA). Following size selection using Pippin Prep (Sage Science, Inc., Beverly, MA, USA), the libraries were amplified for eight cycles using Platinum™ PCR SuperMix high-fidelity and library amplification primer mix supplied with the Ion Xpress™ Plus Fragment Library Kit. Equimolar amounts of the barcoded libraries were pooled (100 pM) for automated template preparation using the Ion 540™ Kit-Chef (Thermo Fisher Scientific, USA) and chip leading with the Ion Chef™ Instrument (Thermo Fisher Scientific, USA). Sequencing was performed with 500 flows which generated 200 bp reads on an Ion S5™ Next-generation sequencing system (Thermo Fisher Scientific, USA).

### 2.7. Sequence and Phylogenetic Analysis of CaPV-Marker Genes

The Sanger sequencing raw reads were assembled using Vector NTI software (Invitrogen) v11.5. Additional CaPVs sequences of each of the targeted genes were retrieved from GenBank for comparative analysis. MEGA X was used to perform multiple sequence alignments for each gene dataset, and for phylogenetic reconstructions. For the neighbor-joining tree construction of the RPO30 and GPCR genes, the evolutionary distances were computed using the Maximum Composite Likelihood method with resampling 1000 times [56]. The phylogenetic trees were visualized and annotated using the Interactive Tree of Life (ITOL) tool [57]. The multiple sequence alignments of the partial EEV glycoprotein and B22R genes were produced and visualized using BioEdit v7.2.5.

### 2.8. Whole-Genome Reconstruction, Annotation, and SNP Analysis of the Lesotho LSDV Isolate

The adaptors were directly removed from the raw read files using the torrent suite software and then quality-filtered using fastq-mcf v1.04.676 using a Phred score cut-off of 20, and the reads of 50 bp to 250 bp lengths were selected (ea-utils). After assessing the read quality with FastQC (v. 011.5), de novo assemblies were performed on a subset of the selected reads using SPAdes (v3.11.1), Unicycler (v0.5.0), and Megahit (v1.2.9). Since the BLAST search identified LSDV_NW-LW (AF409137) as one of the most suitable references, its genome was used for reference-guided assembly of the cleaned raw reads using bowtie2 (2.3.4.1). BCFtools (v1.9) was used to generate Mpileup files from the mapped cleaned raw reads, for variant calling and for consensus calling. Vcfutils.pl (VCFtools v0.1.16) and seqtk (v1.3.106) were used to create consensus sequences with a Phred score of 20. The reference-guided and de novo assemblies were compared after alignment with Mafft (v7.453), the mapping quality was assessed with Qualimap (v.2.2.1), and the alignments were visualized using IGV [58].

The open reading frames (ORFs) of Lesotho LSDV genomes were predicted with GATU using LSDV_NW-LW (AF409137) as a reference genome (Tcherepanov 2006). The complete genome sequences of LSDV_Lesotho_Lac1 and LSDV_Lesotho_490 were submitted to GenBank under the accession numbers PP065789 and PP065788, respectively. To compare the genomes of LSDVs from Lesotho to publicly available LSDVs, the completed dataset with 48 genome sequences was aligned using MAFFT. The single-nucleotide polymorphisms (SNPs), relative to the LSDV RefSeq genome (LSDV_NI-2490, AF325528), were extracted using adegenet package in R. The alignment file was first converted into a genlight object using the function fasta2genlight [59]. The distribution and density of the extracted LSDV SNPs were visualized as heatmap and a discriminant analysis of Principal Component Analysis (DAPCA) was performed. In addition, a phylogenetic network of the aligned LSDV genomes was constructed on the PopART program, using the median-joining network algorithm with epsilon set to zero [60].

## 3. Results

### 3.1. Investigation of the LSD Outbreak and Clinical Signs

**Figure 2:**
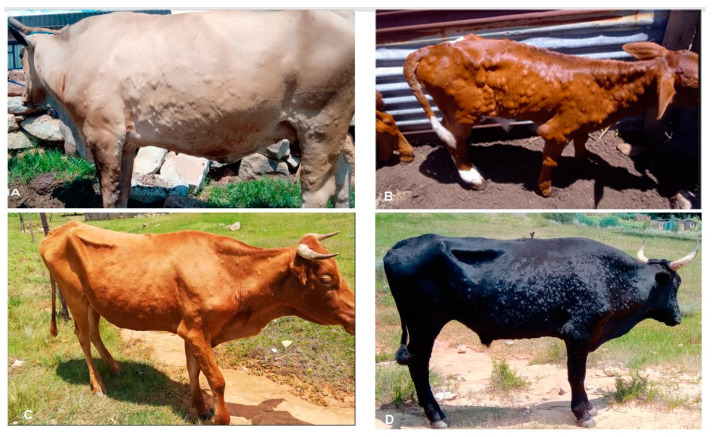
Nodules characteristic of lumpy skin disease on cattle from (**A**) HaMalelu village of Maseru district, (**B**) Sefikeng village in Berea district, (**C**) Ntlama village in Berea district, and (**D**) HaMotemekoane village in Maseru district. The photos were taken after sample collection in February 2022.

A total of 18 cattle from various villages in five districts in the lowlands zone of Lesotho, including Mafeteng, Maseru, Mohales’ Hoek, Leribe, and Berea districts, showed typical LSD clinical signs and symptoms. These clinical signs and symptoms include fever, enlargement of lymph nodes, firm circumscribed nodules on the skin, and ulcerative lesions, particularly on the mucous membranes of the mouth. As shown in Figure 2, cases include different breeds of cattle of all ages and sexes.

### 3.2. Molecular Diagnosis of LSDV

As shown in Table 2, the CaPV genome was detected in all 18 samples analyzed by RT-PCR using the Bowden et al. [54] protocol. Only 11 samples with Cq values less than 25 were sequenced.

### 3.3. Sequence and Phylogenetic Analysis of the Targeted CaPV Genes

**Figure 3:**
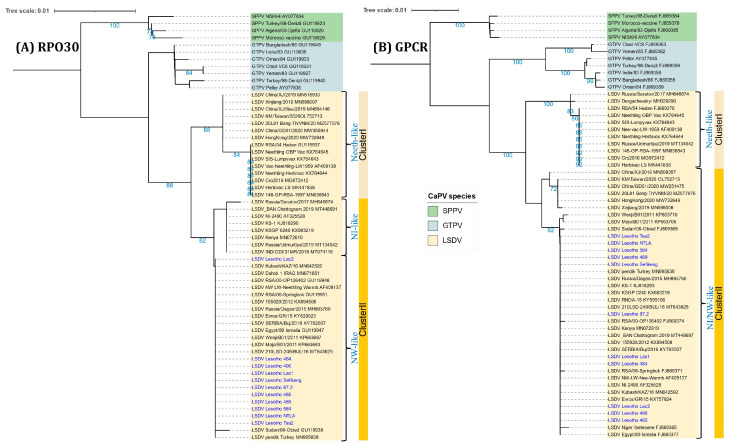
Neighbor-joining tree based on the complete (**A**) RPO30 and (**B**) GPCR gene sequences of CaPVs. The evolutionary distances were computed using the Maximum Composite Likelihood method with 1000 bootstrap replicates on MEGA X and visualized on iTOL. LSDV isolates from Lesotho are written in blue-colored font.

The 11 positive samples with Cq values below 25 were successfully amplified and sequenced for RPO30 (n = 9), GPCR (n = 9), EEV glycoprotein (n = 9), and B22R (n = 4) genes, and as well as two complete genome sequences (Tables S2 and S3. Multiple sequence alignments of the four targets showed that all the LSDV samples from Lesotho are 100% identical. Phylogenetic analysis based on the complete RPO30 and GPCR gene sequences clustered the LSDV samples from Lesotho with NW-like LSDVs within Cluster II, together with the commonly circulating LSDV field isolates encountered in Africa, the Middle East, and Europe (Figure 3).

**Figure 4:**
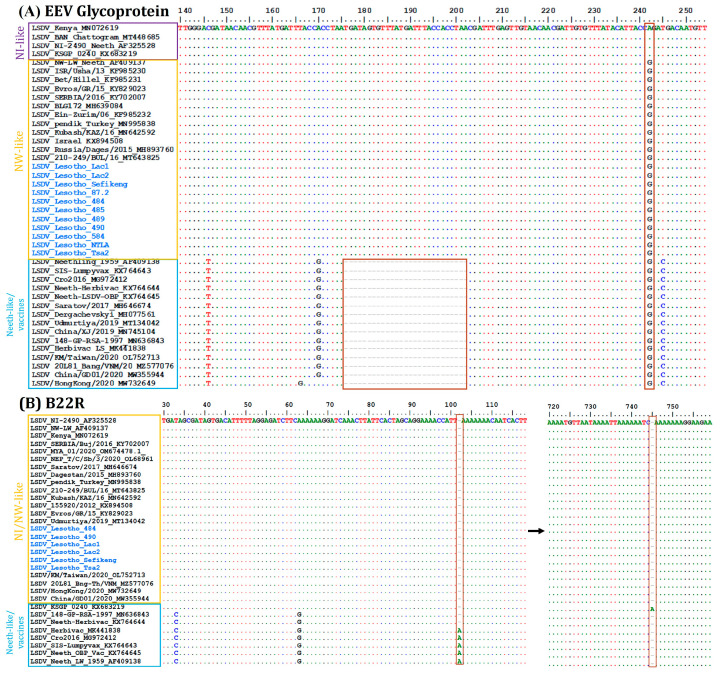
Multiple sequence alignment (Lesotho-isolated highlighted in blue-colored font) of (**A**) partial sequences of EEV glycoprotein gene showing a 27-nucleotide deletion (highlighted in block) that is absent in Lesotho isolates and (**B**) partial sequences of B22R gene showing the nucleotide insertion (in blocks) in LSDV_Neethling and LSDV_KSGPO-240 vaccines that are absent in Lesotho isolates. The dots indicate the identical nucleotides in the alignment.

The multiple sequence alignment of the partial EEV glycoprotein gene and the B22R reiterated that LSDV isolates from Lesotho are identical to commonly circulating LSDV field isolates. In addition, the EEV sequences of Lesotho samples showed a 27-nucleotide insertion similar to LSDV field isolates, differentiating them from LSDV Neethling-derived vaccines. In contrast, the B22R alignment showed a nucleotide insertion at position 102 and 745 only in LSDV Neethling-derived vaccines and LSDV KSGP-0240 vaccine, respectively (Figure 4).

### 3.4. LSDV Whole Genome Analysis

The assembled LSDV genomes of LSDV_Lesotho_Lac1 and LSDV_Lesotho_490 samples were 150,908 bp long, with a mean coverage of 164.59 +/− 47.19 and 605.81 +/− 222.21, respectively. Both genomes were 100% identical and comprised 160 predicted ORFs. A total of 147 of the predicted ORFs were 100% homologous to LSDV_NW-LW isolate (AF409137.1), and the remaining 13 ORFs shared between 77.8 and 99.9% homology. The NCBI nucleotide blast search using the Lesotho LSDV whole genome showed that the Lesotho isolates shared 99.95% nucleotide identity to LSDV_NW-LW reference isolate (AF409137.1), as well as other commonly circulating LSDV field isolates such as LSDV_Pendik (MN995838.1), LSDV_Serbia/2016 (KY702007.1), LSDV_Evros/GR/15 (KY829023.3), and LSDV_210/BUL/16 (MT643825.1).

**Figure 5:**
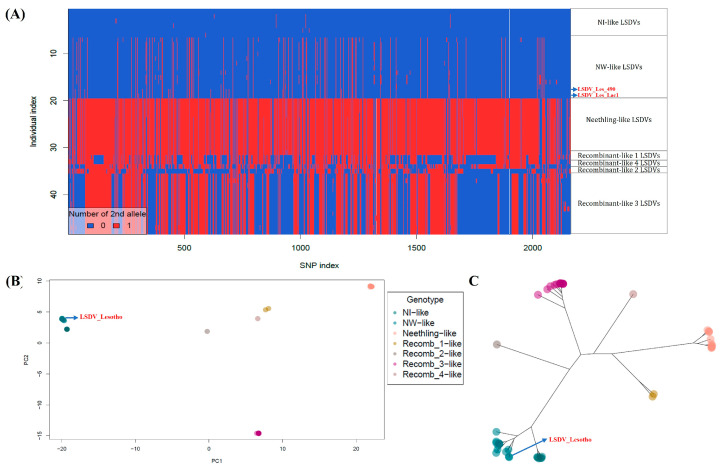
SNP analysis of LSDV whole-genome alignment. Lesotho isolates are labelled in red. (**A**) SNP distribution heatmap, (**B**) PCA-scatter plot, and (**C**) neighbor-joining tree based on the PCA scores of the SNP data.

Furthermore, heatmap and PCA analysis based on the SNP index of the aligned LSDV genomes relative to the LSDV_NI-2490 (AF325528.1) genome revealed the seven clusters of LSDV genotypes: NI-like (ancient LSDVs), NW-like (common field isolates), Neethling-like LSDVs, and four recombinant–LSDV clusters. The heatmap, PCA scatterplot, and the neighbor-joining tree clustered the Lesotho LSDV isolates with the NW-like LSDV genotypes (Figure 5).

**Figure 6:**
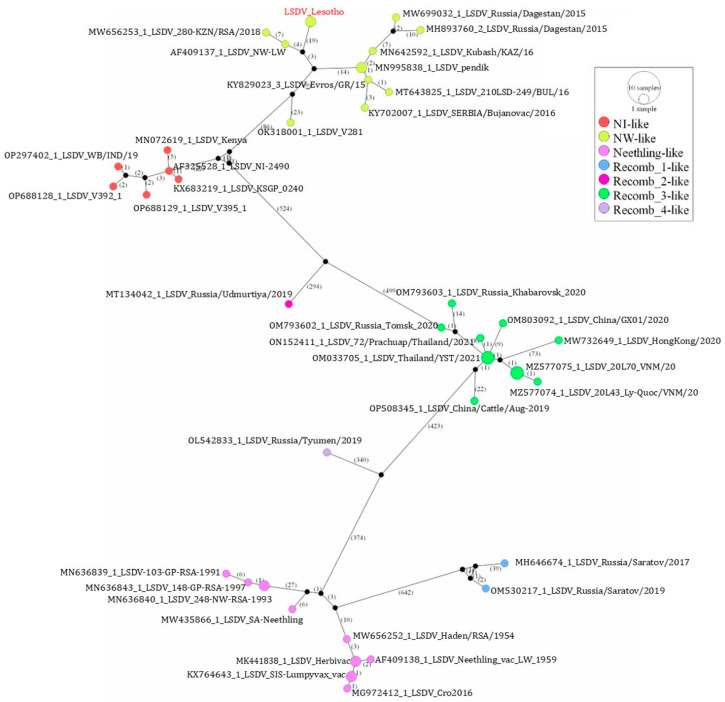
Median-joining phylogenetic network based on LSDV whole-genome sequences using the PopART program, showing the Lesotho LSDVs (in red) clustering with NW-like LSDVs. The number of mutations between each genome is labelled.

In addition, phylogenetic network analysis of the aligned LSDV genomes using PopART software clustered the Lesotho LSDV isolates with NW-like LSDVs (Figure 6).

## 4. Discussion

Lumpy skin disease remains a serious threat to the global livestock industry due to its ability to spread fast and the emergence of new LSDV strains [11,61,62,63]. The virus has been detected in other mammalian wildlife species with typical clinical symptoms, suggesting that the virus has acquired the ability to infect a broader range of hosts and cause disease [64,65]. Since the first outbreak in Lesotho, LSD surveillance was only based on clinical diagnosis. While clinical diagnosis is essential, it is prone to inaccurate diagnosis since LSD shares common symptoms with other diseases, such as pseudo-lumpy skin disease, cutaneous leucosis, and dermatophilus infection [8]. Moreover, the disease may be missed, given that some animals remain asymptomatic while harboring the virus and unknowingly spreading the virus to other susceptible animals [66]. This may make it difficult to control the spread of the virus and adds complexity in understanding and developing effective preventive measures.

In the current study, 18 clinical cases of LSD were identified based on typical clinical signs from the five districts and the capripoxvirus genome was amplified by real-time PCR. Furthermore, the presence of LSDV was successfully confirmed in 11 Lesotho samples by amplifying and sequencing four LSDV genetic markers, namely B22R, GPCR, EEV glycoprotein, and RPO30 [35,36,40]. The results further underscore the importance of clinical diagnosis in the surveillance of LSD. Moreover, phylogenetic analysis based on GPCR and RPO30 gene sequences divided the *Capripox* genus into three groups based on species but also formed two clusters of LSDVs: cluster I and cluster II. All Lesotho LSDV isolates belonged to cluster II and are related to other classical LSDV field strains from various countries, including South Africa, Bulgaria, Kenya, Nigeria, Ethiopia, Sudan, Turkey, and Russia [67]. It is essential to note that the GPCR sequences of the Lesotho LSDVs formed a separate subclade distinct from the recombinant LSDV strain sequence subclade within cluster ll.

B22R is a gene marker used for LSDV confirmation and differentiation between vaccines and field strains [68,69]. The differentiation is based on unique single-nucleotide insertions present in the B22R gene of vaccine strains only, which is absent in field strains [40]. The B22R gene multiple sequence alignments showed 100% identity among Lesotho isolates and the common field isolates circulating in cattle and wildlife populations in the neighboring countries, including South Africa. These findings are consistent with study findings in Nepal and Myanmar, where the B22R gene was applied to differentiate vaccine strains from field type based on the single-nucleotide insertion [40,41]. However, the marker is not capable of distinguishing field strains from recombinant vaccine isolates such as LSDV Russia/Saratova/2017 (MH646674) and LSDV Udmurtya/Russia/2018 (MT134042) [61,62].

The EEV glycoprotein gene is encoded by ORF LSDV126 and plays a significant role in viral attachment to the host cells and the subsequent entry of the virus into these cells [43]. The LSDV EEV glycoprotein gene is one of the most reliable and popular genetic markers used for distinguishing LSDV field strains from vaccine strains in several studies, based on the deletion of 27 bp only in the vaccine strains [70,71]. In the current study, EEV glycoprotein multiple sequence alignment showed two subgroups: field strains with the insertion of 27 bp and vaccine strains (and the recent LSDV recombinants) without the insertion. The findings are consistent with molecular studies conducted on LSDV in Nigeria, Nepal, and Ethiopia [40,72]. Therefore, based on analyses of the four LSDV gene markers, it is evident that all Lesotho isolates likely share common ancestry and belong to the field-type strains. The isolates also exhibit similarities to LSDV isolates from South Africa, Sudan, Kenya, and other countries rather than recombinant strains or vaccine strains [73]. Moreover, the results further strengthen confidence in the reliability and accuracy of LSDV gene markers in the detection and diagnosis of LSDV infections.

The genomes of LSDV_Lesotho_Lac1 (Accession number PP065789) and LSDV_Lesotho_490 (Accession number P065788) are both 150,908bp long and exhibit 100% identity. This remarkable degree of similarity strongly suggests common ancestral origin for these isolates. Furthermore, the results suggest the possibility of long-distance transmission of the disease, since the isolates were collected from Maseru and Leribe districts that are separated by two intermediate districts at distance of over 80 kilometers apart. Both genomes contained a total of 160 predicted ORFs that represent a 95% coding density and encode different proteins that play critical roles in LSDV life cycle and host interaction [43]. Out of 160 ORFs, 147 ORFs exhibited 100% homology, while the remaining 13 ORF homology ranges between 77.8 and 99.9%, with classical field strains isolated from severely infected calves in South Africa in 1999 [74]. The genetic similarity between Lesotho and South Africa LSDV isolates provides evidence of historical linkage, indicating that the virus has been transmitted between the two countries, either through the movement of infected animals or flying vectors, considering the geographical proximity of the two countries. A BLAST analysis using the whole genome revealed that the Lesotho LSDV isolates exhibited 99.95% identity with LSDV_NW_LW (AF409137) from South Africa. These findings agree with those obtained in the ORF-analysis in this study, which further indicates that there was virus transboundary transmission between the two countries, and that Lesotho isolates belong to classical LSDV field strains and not to vaccine or recombinant strains.

Similarly, the Lesotho isolates are also 99.95% identical to other commonly circulating LSDV field isolates in Russia, Serbia, Greece, and Bulgaria. The remarkable similarity observed among these isolates is intriguing, especially considering the substantial geographical separation between the locations where the viruses were isolated. This observation could indicate a limited genetic diversity among field strains, possibly stemming from a shared ancestral lineage or common ancestry of the isolates. Therefore, investigation is needed to determine whether this genetic similarity is due to a recent common ancestor, or it has been maintained over long period of time. Understanding the origins and spread of these isolates could provide valuable insights into the virus’s evolution and potential for future outbreaks.

Interestingly, the phylogenetic network and the heatmap and PCA analysis based on SNP index revealed seven clusters of LSDV genotypes and clustered the Lesotho isolates with NW_LSDVs. This suggests a diverse genetic landscape among LSDV genotypes, with distinct groups representing both ancient and contemporary strains. The presence of recombinant–LSDV clusters further highlights the potential for genetic exchange and evolution within the LSDV population. Further investigation is needed to understand the implications of these clusters on the epidemiology and pathogenicity of LSDVs. Furthermore, the clustering of Lesotho isolates in NW_LSDVs (isolates collected between 2015 and 2018) indicates possible transmission routes and spread of the virus across multiple countries. The next LSDV cluster close to the Lesotho isolates was the NI-like LSDVs, which include the historical strains, while the furthest cluster was the Neethling-like isolates from the 1950s and 1990s.

The presence of field-type LSDV strains in Lesotho and other neighboring countries presents a regional collaboration opportunity in the Southern Africa region, by sharing information, resources, and expertise for effective management of LSD [43,75]. The Lesotho LSDV isolates cluster with the commonly circulating LSDV field isolates; therefore, several vaccines, such as Neethling vaccine strains, LSD SIS Neethling-type strain, and live attenuated LSDV, which have proven effective and safe in the control of LSD in several other countries, including South Africa, can also be used in Lesotho [76]. It is important for the country to consider implementing vaccination programs using these proven vaccines to effectively control and eradicate the disease [77,78,79]. Additionally, since the LSD vaccines used worldwide are mostly live attenuated, the continuous monitoring of the genetic characteristics of isolates can help in investigating LSD outbreaks in vaccinated herds. Further, intensive studies are needed to better understand the epidemiology of this disease in wildlife and other domestic animals.

In conclusion, the prevalence of LSDV was successfully confirmed by observation of clinical signs and use of DNA-based protocols in the current study. The obtained isolates were successfully characterized by molecular techniques, and based on a multi-targeted PCR approach, all identified cases in five districts of Lesotho were caused by LSDV field strains. This is the first study to conduct genetic detection and characterization of LSDV in Lesotho. Further studies are needed to improve understanding of LSDV, including the role of wildlife and domestic animals in the transmission of the virus. Data generated in this study will contribute to improving LSD management practices in Lesotho.

## Figures and Tables

**Table 1:** PCR primers used in this study with sequence, fragment size, and references.

Primer ID	Primer Sequence (3′ to 5′)	Fragment Size bp	Publisher
CpRPO30-OL1F	CAGCTGTTTGTTTACATTTGATTTTT	554	[36]
CpRPO30-OL1R	TCGTATAGAAACAAGCCTTTAATAGA		
CpRPO30-OL2F	TTTGAACACATTTTATTCCAAAAAG	520	
CpRPO30-OL2R	AACCTACATGCATAAACAGAAGC		
CpGPCR-OL1F	TGAAAAATTAATCCATTCTTCTAAACA	617	
CpGPCR-OL1R	TCATGTATTTTATAACGATAATGCAAA		
CpGPCR-OL2F	TTAGCGGTATAATCATTCCAAATA	603	
CpGPCR-OL2R	GCGATGATTATGATGATTATGAAGTG		
CpGPCR-OL3F	CACAATTATATTTCCAAATAATCCAA	684	
CpGPCR-OL3R	TGTACATGTGTAATTTTAATGTTCGTA		
EEVGly-F	ATGGGAATAGTATCTGTTGTATACG	866–931	[35]
EEVGly-R	ATGGGAATAGTATCTGTTGTATACG		
B22R_CaPVFw	TCATTTTCTTCTAGTTCCGACGA	863	[40]
B22R_CaPVRv	TTCGTTGATGATAAATAACTGGAAA		

**Table 2:** Description of the analyzed DNA samples showing the location, collection date, and Cq values.

Sample	Collection Date	Location	Cq Bowden
LSD_Leso_484	16 January 2022	Maseru	23.9
LSD_Leso_490	16 January 2022	Leribe	19.0
LSD_Leso_LAC1	16 January 2022	Maseru	18.3
LSD_Leso_LAC2	16 January 2022	Maseru	21.9
LSD_Leso_Sefikeng	16 January 2021	Berea	22.2
LSD_Leso_87.2	23 February 2022	Mohales’ Hoek	23.5
LSD_Leso_485	17 January 2022	Maseru	24.0
LSD_Leso_489	26 January 2022	Maseru	24.7
LSD_Leso_584	5 February 2022	Maseru	25.0
LSD_Leso_Ntlama	20 February 2021	Berea	24.1
LSD_Leso_Tsakholo	12 March 2021	Mafeteng	23.4
LSD_Leso_506	11 February 2022	Berea	25.0
LSD_Leso_585	5 January 2022	Berea	24.9
LSD_Leso_601	17 March 2022	Mafeteng	26.3
LSD_Leso_88	6 February 2022	Maseru	27.2
LSD_Leso_480	19 January 2021	Leribe	29.0
LSD_Leso_605	20 January 2022	Leribe	28.0
LSD_Leso_604	17 January 2022	Maseru	27.4

## Data Availability

The original contributions presented in the study are included in the article/Supplementary Materials; further inquiries can be directed to the corresponding author.

## Supplementary Materials

The following supporting information can be downloaded at: https://www.mdpi.com/article/10.3390/v16050762/s1, Table S1: Information on specimens, including location, collection date, and geo-coordinates for the period 2022-2022; Table S2: Sequence information including location, isolate name, accession numbers for Whole genome, RPO30, and GPCR gene sequences; Table S3: Sequence information including location, isolate name, accession numbers for EEV glycoprotein and B22R gene sequence.

## References

[1] Chouhan C.S., Parvin M.S., Ali M.Y., Sadekuzzaman M., Chowdhury M.G.A., Ehsan M.A., Islam M.T. (2022). Epidemiology and Economic Impact of Lumpy Skin Disease of Cattle in Mymensingh and Gaibandha Districts of Bangladesh. Transbound. Emerg. Dis..

[2] Suwankitwat N., Songkasupa T., Boonpornprasert P., Sripipattanakul P., Theerawatanasirikul S., Deemagarn T., Suwannaboon M., Arjkumpa O., Buamithup N., Hongsawat A. (2022). Rapid Spread and Genetic Characterisation of a Recently Emerged Recombinant Lumpy Skin Disease Virus in Thailand. Vet. Sci..

[3] Tuppurainen E.S.M., Venter E.H., Shisler J.L., Gari G., Mekonnen G.A., Juleff N., Lyons N.A., De Clercq K., Upton C., Bowden T.R. (2017). Review: Capripoxvirus Diseases: Current Status and Opportunities for Control. Transbound. Emerg. Dis..

[4] (2022). ICTV Taxonomy My Release. https://ictv.global/taxonomy.

[5] Mcinnes C.J., Damon I.K., Smith G.L., Mcfadden G., Isaacs S.N., Roper R.L., Evans D.H., Damaso C.R., Carulei O., Wise L.M. (2023). ICTV Virus Taxonomy Profile: Poxviridae 2023. J. Gen. Virol..

[6] Abeya A., Feyisa B., Gezali A., Derej A. (2018). Review on Epidemiological Aspects and Economic Impact of Lumpy Skin Disease. J. Dairy Vet. Sci..

[7] Roche X., Rozstalnyy A., TagoPacheco D., Kamata A., Pittiglio C., Beltran-alcrudo D., Bisht K., Karki S., Kayamori J., Larfaoui F. (2020). Introduction and Spread of Lumpy Skin Disease in South, East and Southeast Asia: Qualitative Risk Assessment and Management, FAO Animal Production and Health Papers.

[8] Das M., Chowdhury M., Akter S., Mondal A., Uddin M., Rahman M., Rahman M. (2021). An updated review on lumpy skin disease: A perspective of Southeast Asian countries. J. Adv. Biotechnol. Exp. Ther..

[9] Byadovskaya O., Prutnikov P., Shalina K., Babiuk S., Perevozchikova N., Korennoy F., Chvala I., Kononov A., Sprygin A. (2022). The changing epidemiology of lumpy skin disease in Russia since the first introduction from 2015 to 2020. Transbound. Emerg. Dis..

[10] World Organisation for Animal Health (WOAH) (2022). Animal Health Data: Lumpy Skin Disease. World Animal Health Information System..

[11] Dubey A., Ghosh N.S., Gupta A., Singh S. (2023). A review on current epidemiology and molecular studies of lumpy skin disease virus-an emerging worldwide threat to domestic animals. J. Med. Pharm. Allied Sci..

[12] Guyassa C. (2022). Epidemiology and diagnostic methods of lumpy skin disease: A Short Review. Int. J. Vet. Sci. Res..

[13] Liang Z., Yao K., Wang S., Yin J., Ma X., Yin X., Wang X., Sun Y. (2022). Understanding the Research Advances on Lumpy Skin Disease: A Comprehensive Literature Review of Experimental Evidence. Front. Microbiol..

[14] Pandey N., Hopker A., Prajapati G., Rahangdale N., Gore K., Sargison N. (2022). Observations on Presumptive Lumpy Skin Disease in Native Cattle and Asian Water Buffaloes around the Tiger Reserves of the Central Indian Highlands. N. Z. Vet. J..

[15] Akther M., Akter S.H., Sarker S., Aleri J.W., Annandale H., Abraham S., Uddin J.M. (2023). Global Burden of Lumpy Skin Disease, Outbreaks, and Future Challenges. Viruses.

[16] Kumar R., Godara B., Chander Y., Kachhawa J.P., Dedar R.K., Verma A., Riyesh T., Pal Y., Barua S., Tripathi B.N. (2023). Evidence of lumpy skin disease virus infection in camels. Acta Trop..

[17] Mathewos M., Dulo F., Tanga Z., Sombo M. (2022). Clinicopathological and molecular studies on cattle naturally infected with lumpy skin diseases in selected districts of Wolaita Zone, Southern Ethiopia. BMC Vet. Res..

[18] Archana M., D’Souza P.E., Ojha R., Jalali S. (2016). DNA barcoding of flies commonly prevalent in poultry farms of Bengaluru District. J. Entomol. Zool. Stud..

[19] Rouby S.R., Safwat N.M., Hussein K.H., Abdel-Ra’ouf A.M., Madkour B.S., Abdel-Moneim A.S., Hosein H.I. (2021). Lumpy skin disease outbreaks in Egypt during 2017–2018 among sheeppox vaccinated cattle: Epidemiological, pathological, and molecular findings. PLoS ONE.

[20] Mulatu E., Feyisa A. (2018). Review: Lumpy skin disease. J. Vet. Sci. Technol..

[21] Selim A., Manaa E., Khater H. (2021). Seroprevalence and Risk Factors for Lumpy Skin Disease in Cattle in Northern Egypt. Trop. Anim. Health Prod..

[22] Issimov A., Kushaliyev K., Abekeshev N., Molla W., Rametov N., Bayantassova S., Zhanabayev A., Paritova A., Shalmenov M., Ussenbayev A. (2022). Risk factors associated with lumpy skin disease in cattle in West Kazakhstan. Prev. Vet. Med..

[23] Abutarbush S.M., Bayry J. (2017). Lumpy Skin Disease (Knopvelsiekte, Pseudo-Urticaria, Neethling Virus Disease, Exanthema Nodularis Bovis). Emerging and Re-Emerging Infectious Diseases of Livestock.

[24] Sprygin A., Pestova Y., Wallace D.B., Tuppurainen E., Kononov A.V. (2019). Transmission of lumpy skin disease virus: A short review. Virus Res..

[25] Ardestani E.G., Mokhtari A. (2020). Modeling the lumpy skin disease risk probability in central Zagros Mountains of Iran. Prev. Vet. Med..

[26] Bianchini J., Simons X., Humblet M.-F., Saegerman C. (2023). Lumpy Skin Disease: A Systematic Review of Mode of Transmission, Risk of Emergence and Risk Entry Pathway. Viruses.

[27] Datten B., Chaudhary A.A., Sharma S., Singh L., Rawat K.D., Ashraf M.S., Alneghery L.M., Aladwani M.O., Rudayni H.A., Dayal D. (2023). An extensive examination of the warning signs, symptoms, diagnosis, available therapies, and prognosis for lumpy skin disease. Viruses.

[28] Toker E.B., Ates O., Yeşilbağ K. (2022). Inhibition of bovine and ovine capripoxviruses (Lumpy skin disease virus and Sheeppox virus) by ivermectin occurs at different stages of propagation in vitro. Virus Res..

[29] Haig D. (1957). Lumpy skin disease. Bull. Epizoot. Dis. Afr..

[30] Le Goff C., Lamien C.E., Fakhfakh E., Chadeyras A., Aba-Adulugba E., Libeau G., Tuppurainen E., Wallace D.B., Adam T., Silber R. (2009). Capripoxvirus G-protein-coupled chemokine receptor: A host-range gene suitable for virus animal origin discrimination. J. Gen. Virol..

[31] Lamien C.E., Lelenta M., Goger W., Silber R., Tuppurainen E., Matijevic M., Luckins A.G., Diallo A. (2011). Real time PCR method for simultaneous detection, quantitation and differentiation of capripoxviruses. J. Virol. Methods.

[32] Li L., Qi C., Li J., Nan W., Wang Y., Chang X., Chi T., Gong M., Ha D., De J. (2022). Quantitative Real-time PCR Detection and Analysis of a Lumpy Skin Disease Outbreak in Inner Mongolia Autonomous Region, China. Front. Vet. Sci..

[33] Jiang C., Tao D., Geng Y., Yang H., Xu B., Chen Y., Hu C., Chen H., Xie S., Guo A. (2022). Sensitive and Specific Detection of Lumpy Skin Disease Virus in Cattle by Crispr-cas12a Fluorescent Assay Coupled with Recombinase Polymerase Amplification. Genes.

[34] Badhy S.C., Chowdhury M.G.A., Settypalli T.B.K., Cattoli G., Lamien C.E., Fakir M.A.U., Akter S., Osmani M.G., Talukdar F., Begum N. (2021). Molecular Characterization of Lumpy Skin Disease Virus (LSDV) Emerged in Bangladesh Reveals Unique Genetic Features Compared to Contemporary Field Strains. BMC Vet. Res..

[35] Gelaye E., Belay A., Ayelet G., Jenberie S., Yami M., Loitsch A., Tuppurainen E., Grabherr R., Diallo A., Lamien C.E. (2015). Capripox disease in Ethiopia: Genetic differences between field isolates and vaccine strain, and implications for vaccination failure. Antivir. Res..

[36] Samojlović M., Polaček V., Gurjanov V., Lupulović D., Lazić G., Petrović T., Lazić S. (2019). Detection of antibodies against Lumpy skin disease virus by Virus neutralization test and ELISA methods. Acta Vet..

[37] Zewdie G., Mammo B., Gelaye E., Getachew B., Bayssa B. (2019). Isolation, molecular characterisation and vaccine effectiveness study of lumpy skin disease virus in selected dairy farms of Central Ethiopia. J. Biol. Agric. Healthc..

[38] Heine H.G., Stevens M.P., Foord A.J., Boyle D.B. (1999). A Capripoxvirus detection PCR and antibody ELISA based on the major antigen p32, the homolog of the vaccinia virus H3L gene. J. Immunol. Methods.

[39] Koirala P., Meki I.K., Maharjan M., Settypalli B.K., Manandhar S., Yadav S.K., Cattoli G., Lamien C.E. (2022). Molecular Characterization of the 2020 Outbreak of Lumpy Skin Disease in Nepal. Microorganisms.

[40] Maw M.T., Khin M.M., Hadrill D., Meki I.K., Settypalli T.B.K., Kyin M.M., Myint W.W., Thein W.Z., Aye O., Palamara E. (2022). First Report of Lumpy Skin Disease in Myanmar and Molecular Analysis of the Field Virus Isolates. Microorganisms.

[41] Pareek C.S., Smoczynski R., Tretyn A. (2011). Sequencing Technologies and Genome Sequencing. J. Appl. Genet..

[42] Tulman E.R., Afonso C.L., Lu Z., Zsak L., Kutish G.F., Rock D.L. (2001). Genome of Lumpy Skin Disease Virus. J. Virol..

[43] Breman F.C., Haegeman A., Krešić N., Philips W., De Regge N. (2023). Lumpy Skin Disease Virus Genome Sequence Analysis: Putative Spatio-temporal Epidemiology, Single Gene Versus Whole Genome Phylogeny and Genomic Evolution. Viruses.

[44] Giani A.M., Gallo G.R., Gianfranceschi L., Formenti G. (2020). Long walk to genomics: History and current approaches to genome sequencing and assembly. Comput. Struct. Biotechnol. J..

[45] Kumar A., Venkatesan G., Kushwaha A., Poulinlu G., Saha T., Ramakrishnan M.A., Dhar P., Kumar G.S., Singh R.K. (2023). Genomic characterization of Lumpy Skin Disease virus (LSDV) from India: Circulation of Kenyan-like LSDV strains with unique kelch-like proteins. Acta Trop..

[46] Alkhamis M.A., Vanderwaal K. (2016). Spatial and Temporal Epidemiology of Lumpy Skin Disease in the Middle East, 2012–2015. Front. Vet. Sci..

[47] Machado G., Korennoy F., Alvarez J., Picasso-Risso C., Perez A., Vanderwaal K. (2019). Mapping Changes in the Spatiotemporal Distribution of Lumpy Skin Disease Virus. Transbound. Emerg. Dis..

[48] Mathijs E., Vandenbussche F., Haegeman A., King A., Nthangeni B., Potgieter C., Maartens L., Borm S.V., Clercq K.D. (2016). Complete Genome Sequences of the Neethling-Like Lumpy Skin Disease Virus Strains Obtained Directly from Three Commercial Live Attenuated Vaccines. Genome Announc..

[49] Mafirakureva P., Saidi B., Mbanga J. (2017). Incidence and Molecular Characterisation of Lumpy Skin Disease Virus in Zimbabwe Using the P32 Gene. Trop. Anim. Health Prod..

[50] Molini U., Boshoff E., Niel A.P., Phillips J., Khaiseb S., Settypalli T.B.K., Dundon W.G., Cattoli G., Lamien C.E. (2021). Detection of Lumpy Skin Disease Virus in an Asymptomatic Eland (taurotragus Oryx) in Namibia. J. Wildl. Dis..

[51] Motlhale M. (2016). Heterologous Expression of LSDV Immunogenic Epitopes as TMV Coat Protein Fusions in *Nicotiana benthamiana* Plants. Master’s Thesis.

[52] World Organization of Animal Health (WOAH) World Animal Health Information System—Lumpy Skin Disease. https://web.oie.int/hs2/zi_pays_mald.asp?c_pays=112&annee=2004&c_mald=8.

[53] Bowden T.R., Babiuk S.L., Parkyn G.R., Copps J.S., Boyle D.B. (2008). Capripoxvirus tissue tropism and shedding: A quantitative study in experimentally infected sheep and goats. Virology.

[54] Sendow I., Meki I.K., Dharmayanti N.L.P.I., Hoerudin H., Ratnawati A., Settypalli T.B.K., Ahmed H.O., Nuradji H., Saepulloh M., Adji R.S. (2024). Molecular characterization of recombinant LSDV isolates from 2022 outbreak in Indonesia through phylogenetic networks and whole-genome SNP-based analysis. BMC Genom..

[55] Kumar K. (2018). Classical and Bayesian estimation in log-logistic distribution under random censoring. Int. J. Syst. Assur. Eng. Manag..

[56] Letunic I., Bork P. (2021). Interactive Tree of Life (itol) V5: An Online Tool for Phylogenetic Tree Display and Annotation. Nucleic Acids Res..

[57] Robinson J.T., Thorvaldsdóttir H., Turner D., Mesirov J.P. (2023). igv. js: An embeddable JavaScript implementation of the Integrative Genomics Viewer (IGV). Bioinformatics.

[58] Jombart T., Ahmed I. (2011). Adegenet 1.3-1: New Tools for the Analysis of Genome-wide SNP Data. Bioinformatics.

[59] Leigh J.W., Bryant D. (2015). POPART: Full-feature software for haplotype network construction. Methods Ecol. Evol..

[60] Aleksandr K., Olga B., David W.B., Pavel P., Yana P., Svetlana K., Alexander N., Vladimir R., Dmitriy L., Alexander S. (2020). Non-vector-borne transmission of lumpy skin disease virus. Sci. Rep..

[61] Sprygin A., Pestova Y., Bjadovskaya O., Prutnikov P., Zinyakov N., Kononova S., Ruchnova O., Lozovoy D., Chvala I., Kononov A. (2020). Evidence of recombination of vaccine strains of lumpy skin disease virus with field strains, causing disease. PLoS ONE.

[62] Shumilova I., Sprygin A., Mazloum A., Pronin V., Byadovskaya O., Babiuk S., Donnik I., Chvala I. (2023). Comparison of gross pathology between classical and recombinant lumpy skin disease viruses. Viruses.

[63] Ochwo S., VanderWaal K., Munsey A., Ndekezi C., Mwebe R., Okurut A.R.A., Nantima N., Mwiine F.N. (2018). Spatial and temporal distribution of lumpy skin disease outbreaks in Uganda (2002–2016). BMC Vet. Res..

[64] Dao T.D., Tran L.H., Nguyen H.D., Hoang T.T., Nguyen G.H., Tran K.V.D., Nguyen H.X., Van Dong H., Bui A.N., Bui V.N. (2022). Characterization of Lumpy skin disease virus isolated from a giraffe in Vietnam. Transbound. Emerg. Dis..

[65] Haegeman A., Sohier C., Mostin L., De Leeuw I., Van Campe W., Philips W., De Regge N., De Clercq K. (2023). Evidence of lumpy skin disease virus transmission from subclinically infected cattle by Stomoxys calcitrans. Viruses.

[66] Mathijs E., Vandenbussche F., Ivanova E., Haegeman A., Aerts L., De Leeuw I., Van Borm S., De Clercq K. (2020). Complete coding sequence of a lumpy skin disease virus from an outbreak in Bulgaria in 2016. Microbiol. Resour. Announc..

[67] Van Schalkwyk A., Kara P., Ebersohn K., Mather A., Annandale C.H., Venter E.H., Wallace D.B. (2020). Potential link of single nucleotide polymorphisms to virulence of vaccine-associated field strains of lumpy skin disease virus in South Africa. Transbound. Emerg. Dis..

[68] Krotova A., Mazloum A., van Schalkwyk A., Prokhvatilova L., Gubenko O., Byadovskaya O., Chvala I., Sprygin A. (2022). The characterization and differentiation of recombinant lumpy skin disease isolates using a region within ORF134. Appl. Microbiol..

[69] (Menasherow S., Rubinstein-Giuni M., Kovtunenko A., Eyngor Y., Fridgut O., Rotenberg D., Khinich Y., Stram Y. (2014). Development of an assay to differentiate between virulent and vaccine strains of lumpy skin disease virus (LSDV). J. Virol. Methods.

[70] Zaghloul M., Azooz M., Ali S., Soliman H., Sayed M., Kafafy M., Morsy A. (2022). Sequencing and phylogenetic analysis of GPCR, RPO30, P32 and EEV glycoprotein genes of lumpy skin disease virus recent isolates in Egypt. J. Virol. Sci. Spec..

[71] Chibssa T.R., Sombo M., Lichoti J.K., Adam T.I.B., Liu Y., Elraouf Y.A., Grabherr R., Settypalli T.B.K., Berguido F.J., Loitsch A. (2021). Molecular analysis of East African lumpy skin disease viruses reveals a mixed isolate with features of both vaccine and field isolates. Microorganisms.

[72] van Schalkwyk A., Kara P., Heath L. (2022). Phylogenomic characterization of historic lumpy skin disease virus isolates from South Africa. Arch. Virol..

[73] Kara P., Afonso C., Wallace D., Kutish G., Abolnik C., Lu Z., Vreede F., Taljaard L., Zsak A., Viljoen G.J. (2003). Comparative sequence analysis of the South African vaccine strain and two virulent field isolates of lumpy skin disease virus. Arch. Virol..

[74] Molini U., Aikukutu G., Khaiseb S., Haindongo N.N., Lilungwe A.C., Cattoli G., Dundon W.G., Lamien C.E. (2018). Molecular characterization of lumpy skin disease virus in Namibia, 2017. Arch. Virol..

[75] Modise B.M., Settypalli T.B.K., Kgotlele T., Xue D., Ntesang K., Kumile K., Naletoski I., Nyange J.F., Thanda C., Macheng K.N. (2021). First molecular characterization of poxviruses in cattle, sheep, and goats in Botswana. Virol. J..

[76] Haegeman A., De Leeuw I., Mostin L., Campe W.V., Aerts L., Venter E., Tuppurainen E., Saegerman C., De Clerk K. (2021). Comparative evaluation of lumpy skin disease virus-based live attenuated vaccines. Vaccines.

[77] Tuppurainen E., Dietze K., Wolff J., Bergmann H., Beltran-Alcrudo D., Fahrion A., Lamien C.E., Busch F., Sauter-Louis C., Conraths F.J. (2021). Vaccines and vaccination against lumpy skin disease. Vaccines.

[78] Wolff J., Moritz T., Schlottau K., Hoffmann D., Beer M., Hoffmann B. (2020). Development of a safe and highly efficient inactivated vaccine candidate against lumpy skin disease virus. Vaccines.

[79] Matsiela M.S., Naicker L., Dibakwane V.S., Ntombela N., Khoza T., Mokoena N. (2022). Improved safety profile of inactivated Neethling strain of the lumpy skin disease vaccine. Vaccine X.

