# Supplementary material for: Genetic Characterization of Lumpy Skin Disease Viruses Circulating in Lesotho Cattle
Source: Viruses. 2024 May 11;16(5):762. doi: 10.3390/v16050762 (PMC11125814; doi:10.3390/v16050762)
Supplement: Supplementary file 1 [file viruses-16-00762-s001.zip › viruses-2988556-supplementary.pdf]

Table S1: Information on specimens, including location, collection date, and geo-coordinates for the period 2022-2022

| Sample | Collected date | District | Village | Latitude | Longitude |
|---|---|---|---|---|---|
| LSD_Leso_Tsakholo | 12/3/2021 | Mafeteng | Tsakholo | -29.6498 | 27.1552844 |
| LSD_Leso_585 | 5/1/2022 | Berea | Mokhethoaneng | -29.23466 | 27.564492 |
| LSD_Leso_584 | 5/2/2022 | Maseru | mantsebo | -29.4655 | 27.5055461 |
| LSD_Leso_88 | 6/2/2022 | Maseru | Ha Malelu | -29.56243 | 27.394117 |
| LSD_Leso_506 | 11/2/2022 | Berea | Foso | -29.26961 | 27.592152 |
| LSD_Leso_Sefikeng | 16/01/2021 | Berea | Sefikeng | -29.28248 | 27.810202 |
| LSD_Leso_484 | 16/01/2022 | Maseru | Ha Motemekoane | -29.50097 | 27.543678 |
| LSD_Leso_490 | 16/01/2022 | Leribe | Liphakoeng | -28.93504 | 27.825098 |
| LSD_Leso_LAC1 | 16/01/2022 | Maseru | LAC | -29.29119 | 27.502982 |
| LSD_Leso_LAC2 | 16/01/2022 | Maseru | LAC | -29.29119 | 27.502982 |
| LSD_Leso_485 | 17/01/2022 | Maseru | Ha Mmajane | -29.54516 | 27.453774 |
| LSD_Leso_604 | 17/01/2022 | Maseru | Ha Mokhele | -29.55582 | 27.466205 |
| LSD_Leso_601 | 17/03/2022 | Mafeteng | Matelile | -29.81553 | 27.4986183 |
| LSD_Leso_480 | 19/01/2021 | Leribe | Liphakoeng | -28.93504 | 27.825098 |
| LSD_Leso_605 | 20/01/2022 | Leribe | Liphakoeng | -28.95184 | 27.854111 |
| LSD_Leso_Ntlama | 20/02/2021 | Berea | Ntlama | -29.1722 | 27.8731863 |
| LSD_Leso_87.2 | 23/02/2022 | Mohales' Hoek | Lengopeng la-ntja | -29.31089 | 27.495247 |
| LSD_Leso_489 | 26/01/2022 | Maseru | | -29.44725 | 27.505546 |

Table S2: Sequence information including location, isolate name, accession numbers for Whole genome, RPO30, and GPCR gene sequences

| Item No. | Location | Name of Isolate | Accession No. | Gene sequence |
|---|---|---|---|---|
| 1 | Leribe | LSDV_Leso_490 | PP065788 | Whole genome |
| 2 | Maseru | LSDV_Leso_Lac1 | PP065789 | Whole genome |
| 3 | Mohales Hoek | LSDV_Leso_87.2 | PP065790 | RPO30 |
| 4 | Maseru | LSDV_Leso_484 | PP065791 | RPO30 |
| 5 | Maseru | LSDV_Leso_485 | PP065792 | RPO30 |
| 6 | Maseru | LSDV_Leso_489 | PP065793 | RPO30 |
| 7 | Maseru | LSDV_Leso_584 | PP065794 | RPO30 |
| 8 | Maseru | LSDV_Leso_Lac2 | PP065795 | RPO30 |
| 9 | Berea | LSDV_Leso_Sefikeng | PP065796 | RPO30 |
| 10 | Berea | LSDV_Leso_NTLA | PP065797 | RPO30 |
| 12 | Mafeteng | LSDV_Leso_Tsa2 | PP065798 | RPO30 |
| 13 | Mohales' Hoek | LSDV_Leso_87.2 | PP065799 | GPCR |
| 14 | Maseru | LSDV_Leso_484 | PP065800 | GPCR |
| 15 | Maseru | LSDV_Leso_485 | PP065801 | GPCR |
| 16 | Maseru | LSDV_Leso_489 | PP065802 | GPCR |
| 17 | Maseru | LSDV_Leso_584 | PP065803 | GPCR |
| 18 | Maseru | LSDV_Leso_Lac2 | PP065804 | GPCR |
| 19 | Berea | LSDV_Leso_Sefikeng | PP065805 | GPCR |
| 20 | Berea | LSDV_Leso_NTLA | PP065806 | GPCR |
| 21 | Mafeteng | LSDV_Leso_Tsa2 | PP065807 | GPCR |

Table S3: Sequence information including location, isolate name, accession numbers for EEV glycoprotein and B22R gene sequence

| Item No. | Location | Name of Isolate | Accession No. | Gene sequence |
|---|---|---|---|---|
| 1 | Mohales' Hoek | LSDV_Leso_87.2 | PP065808 | EEV glycoprotein |
| 2 | Maseru | LSDV_Leso_484 | PP065809 | EEV glycoprotein |
| 3 | Maseru | LSDV_Leso_485 | PP065810 | EEV glycoprotein |
| 4 | Maseru | LSDV_Leso_489 | PP065811 | EEV glycoprotein |
| 5 | Maseru | LSDV_Leso_584 | PP065812 | EEV glycoprotein |
| 6 | Maseru | LSDV_Leso_Lac2 | PP065813 | EEV glycoprotein |
| 7 | Berea | LSDV_Leso_Sefikeng | PP065814 | EEV glycoprotein |
| 8 | Berea | LSDV_Leso_NTLA | PP065815 | EEV glycoprotein |
| 9 | Mafeteng | LSDV_Leso_Tsa2 | PP065816 | EEV glycoprotein |
| 10 | Maseru | LSDV_Leso_484 | PP065817 | B22R |
| 11 | Maseru | LSDV_Leso_Lac2 | PP065818 | B22R |
| 12 | Berea | LSDV_Leso_Sefikeng | PP065819 | B22R |
| 13 | Mafeteng | LSDV_Leso_Tsa2 | PP065820 | B22R |
